# Tumour characteristics and survivorship in a cohort of breast cancer: the MCC-Spain study

**DOI:** 10.1007/s10549-020-05600-x

**Published:** 2020-04-30

**Authors:** Inés Gómez-Acebo, Trinidad Dierssen-Sotos, Camilo Palazuelos-Calderón, Beatriz Pérez-Gómez, Pilar Amiano, Marcela Guevara, Antonio J. Molina, Laia Domingo, María Fernández-Ortiz, Victor Moreno, Juan Alguacil, Guillermo Fernández-Tardón, Josefa Ibáñez, Rafael Marcos-Gragera, Marian Diaz-Santos, M. Henar Alonso, Jessica Alonso-Molero, Gemma Castaño-Vinyals, Andrés García Palomo, Eva Ardanaz, Amaia Molinuevo, Nuria Aragonés, Manolis Kogevinas, Marina Pollán, Javier Llorca

**Affiliations:** 1 Consortium for Biomedical Research in Epidemiology and Public Health (CIBER Epidemiología y Salud Pública, CIBERESP), Madrid, Spain; 2 University of Cantabria – IDIVAL, Santander, Spain; 3 Cancer and Environmental Epidemiology Unit, National Center for Epidemiology, Carlos III Institute of Health, Madrid, Spain; 4 Epidemiology Section, Department of Health, Public Health Division, Madrid, Spain; 5 Public Health Division of Gipuzkoa, BioDonostia Research Institute, San Sebastian, Spain; 6 Navarra Public Health Institute, Pamplona, Spain; 7 IdiSNA, Navarra Institute for Health Research, Pamplona, Spain; 8 Instituto de Biomedicina (IBIOMED), Universidad de León, León, Spain; 9 Department of Epidemiology and Evaluation, IMIM (Hospital del Mar Medical Research Institute), Passeig Marítim, 25-29, 08003 Barcelona, Spain; 10 Research Network On Health Services in Chronic Diseases (REDISSEC), Barcelona, Spain; 11 Oncology Data Analytics Program, Hospitalet de Llobregat, Catalan Institute of Oncology (ICO), Barcelona, Spain; 12 Colorectal Cancer Group, ONCOBELL Program, Bellvitge Biomedical Research Institute (IDIBELL), Hospitalet de Llobregat, Barcelona, Spain; 13 Department of Clinical Sciences, Faculty of Medicine, University of Barcelona, Barcelona, Spain; 14 Centro de Investigación en Recursos Naturales, Salud y Medio Ambiente (RENSMA), Universidad de Huelva, Huelva, Spain; 15 Instituto de Investigación Sanitaria del Principado de Asturias-ISPA, UNIOVI and CIBERESP, Oviedo, Spain; 16 Cancer and Public Health Area, FISABIO - Public Health, Valencia, Spain; 17 General Directorate of Public Health, Valencia, Valencian Community Spain; 18 Epidemiology Unit and Girona Cancer Registry. Oncology Coordination Plan, Department of Health, Autonomous Government of Catalonia, Catalan Institute of Oncology, Av. França, s/n, 17007 Girona, Spain; 19 Descriptive Epidemiology, Genetics and Cancer Prevention Group, Biomedical Research Institute (IDIBGI), C/ Dr. Castany, s/n, 17190 Salt, Spain; 20 Centro de Investigación en Recursos Naturales, Salud y Medio Ambiente (RENSMA), Universidad de Huelva, Huelva, Spain; 21 ISGlobal, Barcelona, Spain; 22 IMIM (Hospital del Mar Medical Research Institute), Barcelona, Spain; 23 Universitat Pompeu Fabra (UPF), Barcelona, Spain; 24 Servicio de Oncología, Complejo Asistencial Universitario de León, León, Spain; 25 Facultad de Medicina, Universidad de Cantabria, Avda. Herrera Oria s/n, 39011 Santander, Spain

**Keywords:** Cohort, Epidemiology, Breast cancer, Relative survival, MCC-spain

## Abstract

**Purpose:**

The objective of this study is to analyse the relative survival with breast cancer in women diagnosed after new treatments were generalised and to ascertain the current effect that tumour characteristics such as grade, stage or subtype have on survival as well as the new AJCC-pathological prognostic score.

**Methods:**

The breast cancer MCC-Spain follow-up study is a prospective cohort study of 1685 incident breast cancer cases. Women between 20 and 85 years old were recruited between the years 2008 and 2013 in 18 hospitals located in 10 Spanish provinces and they have been followed until 2017/2018. Relative survival was estimated after 3, 5 and 8 years of follow-up using Ederer II method. In addition, Weibull regression adjusted by age, hospital, grade and stage was used to investigate prognosis factors.

**Results:**

Among components of TNM staging system, tumour size greater than 50 mm (i.e. T3 or T4) more than doubled the risk of dying, while N3 nodal involvement and presence of metastasis had a huge effect on mortality. The AJCC pathological prognostic score strongly correlated with survival; thus, hazard ratios increased as the score rose, being 2.31, 4.00, 4.94, 7.92, 2.26, 14.9 and 58.9 for scores IB, IIA, IIB, IIIA, IIIB, IIIC and IV, respectively.

**Conclusion:**

Both TNM staging and histological/molecular biomarkers are associated with overall survival in Spanish women with breast cancer; when both are combined in the AJCC pathological prognosis score, the prognostic value improved with risk indices that increased rapidly as the pathological prognosis score increased

**Electronic supplementary material:**

The online version of this article (10.1007/s10549-020-05600-x) contains supplementary material, which is available to authorised users.

## Introduction

Breast cancer (BC) is the most frequent cancer and the fourth cause of death by cancer in women in developed countries [1]. Rates of survival with breast cancer have increased significantly all over the world in the past decades [2]. In Spain, it has been estimated that 32,825 new BC cases were diagnosed in 2018, with an incidence rate (Age-standardised [World]) of 75.4 cases per 100,000 women-years [3]. Main factors associated with prognosis in breast cancer are stage (enclosing tumour size and local infiltration, lymph nodes and metastasis), tumour grade of differentiation, histological type and presence/absence of hormone and Her2/neu receptors [2].

Although paclitaxel has been available since 1993, breast cancer treatment has considerably improved from 1995 onwards as conservative surgery has become widely performed. Systemic therapy has switched from CMF (cyclophosphamide, methotrexate and 5-fluorouracil) to regimens containing anthracycline [4], routine searches for oestrogen and progesterone receptors has allowed for the introduction of tamoxifen, as well as identification of tumours positive to Her2/neu has enabled targeted therapy with trastuzumab [5], which was authorised in the European Union on August 28, 2000. In this regard, identification of intrinsic subtypes (i.e. luminal A, luminal B, Her2-positive and basal-like) has allowed for agreement in general recommendations for first-line therapy in early-stage breast cancer [6]. Survival has thus enhanced and 5-year survival is now around 85% [7–9]. In spite of the improvement in systemic therapy, tumour stage remains a key prognosis factor in breast cancer patients [10], which reinforces the relevance of early diagnosis. The American Joint Committee for Cancer (AJCC) has recently suggested a pathological prognostic score combining tumour stage, grade and hormonal/Her2 receptors [11], which has been proved effective as survival predictor [12, 13].

Studies carried out in population-based cancer registries covering 17% of the Spanish population reported that women with BC diagnosed in Spain had 86.0% 5-year age-adjusted relative survival if diagnosed between 1995 and 1999 [7, 14] with no improvements for women diagnosed from 2000 to 2007 [7]. That study, however, included women diagnosed well before the extension of anthracycline-based chemotherapy and the introduction of endocrine therapy with tamoxifen and anti-Her2 treatment with trastuzumab. The main goal in this study is to analyse the relative survival with BC in women diagnosed in 2008–2013—after new treatments were generalised—and to ascertain the current effect that tumour characteristics such as grade, stage or subtype have on survival as well as the new AJCC-pathological prognostic score.

## Methods

### Setting and patients

The breast cancer MCC-Spain follow-up study is a prospective cohort study of incident breast cancer cases diagnosed between 2008 and 2013 in 18 hospitals located in 10 Spanish provinces (Asturias, Barcelona, Cantabria, Girona, Gipuzkoa, Huelva, León, Madrid, Navarra and Valencia). 1738 women between 20 and 85 years old with newly diagnosed primary breast cancer were recruited in the context of a case–control study. The identification of incident cases was carried out by active search through periodic visits to the relevant hospital departments (i.e. gynaecology, oncology, general surgery, radiotherapy and pathology departments). All the cases that were included had histological confirmation and included all the malignant BC (International Classification of Diseases 10th Revision (ICD-10) [15] code C50) and frequent breast cancers in situ (ICD-10: D05.1, D05.7), without a malignant history of BC, diagnosed between 2008 and 2013 in the selected hospitals. Patients were residing in the catchment areas of hospitals for at least 6 months before recruitment. A detailed description of methods and sample characteristics has been published elsewhere [16, 17] and then, 98% recruited patients have been followed until 2017/2018 (*N* = 1685). Only patients signing an informed consent were recruited; the informed consent also asked them for their permission for later consulting their medical records during the follow-up.

### Information obtained at recruitment

When the patients were recruited, medical records were reviewed in order to gather information on age, date of diagnosis, tumour characteristics, including tumour size, lymph nodes, presence of metastases, grade of differentiation. Furthermore, oestrogen (ER), progesterone (PgR) and human epidermal growth factor receptor 2 (HER2) status were assessed and then classified as positive (+), negative (−) and unknown. Specifically, ER and PgR were considered positive if 1% or more of neoplastic cells showed nuclear immunohistochemical (IHC) staining. HER2 was considered positive (overexpressed) if graded 3 + on IHC performed, and all other grades (0 to 2 +) were considered negative unless fluorescence in situ hybridization of 2 + cases confirmed increased gene copy number. Molecular subtypes of breast carcinoma is classified into luminal A (ER + and / or PR + , HER2-, Ki-67 < 14%), luminal B (ER + and / or PR + , HER2-, or ER + and / or PR + , HER2-, Ki-67 ≥ 14%), HER2 + (ER-, PR-, HER2 +) and basal-like or triple negative is a tumour which does not meet any pathologic criteria for positivity of oestrogen receptor, progesterone receptor, or ERBB2 (ER-, PR- and HER2-) [6]. Tumour stage was based on the AJCC standards [18]. Tumour size was classified as T1: ≤ 2 cm, T2: > 2 cm and ≤ 5 cm, T3: > 5 cm, T4: any size with direct extension to chest wall and/or skin; lymph node involvement was classified as N0: no pathologically proven positive lymph nodes, N1: 1–3 positive nodes, N2: 4–9 positive nodes, N3: ≥ 10 positive nodes. Tumour grade was categorised as I: well differentiated, II: moderately differentiated, III: poorly differentiated, according to the modified Bloom and Richardson grading system [19]; when Bloom and Richardson grade was unavailable, other similar classifications were used as it has been proved they are highly reliable [20]. First-line therapy was also recorded, including type of surgery (conservative or mastectomy), margins (free or invaded), chemotherapy regime, radiotherapy, endocrine therapy, Her2-targeted therapy; for all systemic therapies, whether they were adjuvant or neoadjuvant was also recorded.

### Follow-up

In 2017 and 2018, medical records were reviewed to obtain the last date the patient was attended to in t the health system and to ascertain current patient’s vital status. For patients that had not have been at hospital in the 3 months before their medical record review, the National Death Index (Índice Nacional de Defunciones) was consulted [21]. This index is a database recording date of death for all people who have died in Spain; it is hosted on the Ministry of Health web page and can only be accessed with specific authorisation for research purposes. Last date of search in the National Death Index was in December 2018.

### Statistical analysis

Deaths by any cause were considered events and patients alive in their last contact with hospital and not included in the National Death Index were considered censored. Time of follow-up was considered as the time from diagnosis to death or to the last contact in hospital in alive patients. Relative survival, defined as the ratio of the observed survival in our study population and the expected survival in general population Spanish women with the same age as cases in the year of diagnosis, was estimated using Ederer II method [22]; for this purpose, age-specific mortality rates from 2007 to 2018 obtained from the Spanish National Institute for Statistics (INE) were used as reference. 3-, 5- and 8-year survival Kaplan–Meier estimates were obtained. Weibull regression adjusting by age, hospital, grade and stage was used to investigate prognosis factors; its results are displayed as hazard ratios with their 95% confidence intervals. Groups that included less than 25 people were excluded from this analysis. Stata 14/SE package was used in the analyses.

## Results

### Characteristics of patients

We followed 1685 women out of 1738 breast cancer patients (98%), who had primary breast cancer between 2007 and 2013. Patient-years of follow-up were 10,931 person-years. The maximum period for breast cancer follow-up was nine and a half years (from 13th July 2007 to 22nd March 2018) and 206 patients died in the follow-up; 5-year survival was 90.7%.

The mean age at diagnosis was 56.5 ± 12.6-year-old and 65% were postmenopausal. Other main characteristics of the patients are shown in supplementary Table 1.

**Table 1:** Relative survival probability 3, 5 and 8 years after breast cancer diagnosis according to tumour characteristics

Variable	Category	*N* (%)	3-year	5-year	8-year
			Survival (95% CI)	Survival (95% CI)	Survival (95% CI)
Histology	Ductal	1276 (75.73)	97 (95–98)	93 (92–95)	91 (88–93)
	Lobular	110 (6.53)	95 (88–98)	89 (80–94)	87 (76–94)
	Other	299 (17.74)	100 (98–101)	97 (94–99)	97 (92–100)
Tumour size	T1	876 (51.99)	100 (99–101)	98 (97–99)	97 (95–99)
	T2	424 (25.16)	96 (93–98)	91 (87–94)	87 (81–91)
	T3	73 (4.33)	88 (77–94)	75 (63–85)	71 (58–81)
	T4	39 (2.31)	74 (56–86)	59 (41–74)	59 (40–75)
	Tis	109 (6.47)	101 (101–101)	102 (102–102)	100 (91–103)
	Missing	141 (8.37)	93 (86–97)	87 (80–93)	81 (69–90)
Node infiltration	N0	877 (52.05)	100 (99–100)	97 (95–99)	98 (96–100)
	N1	441 (26.17)	97 (95–99)	94 (91–96)	90 (85–94)
	N2	142 (8.43)	93 (86–97)	85 (77–91)	78 (68–86)
	N3	49 (2.91)	81 (67–91)	77 (61–87)	61 (40–78)
	Missing	176 (10.45)	92 (86–96)	88 (81–93)	79 (69–87)
Tumour stage	0	112 (6.65)	100 (95–101)	101 (96–102)	98 (89–102)
	I	594 (35.25)	101 (99–101)	99 (97–101)	100 (97–102)
	II	498 (29.55)	98 (96–100)	95 (92–97)	93 (88–96)
	III	187 (11.10)	94 (89–97)	88 (82–93)	81 (72–88)
	IV	42 (2.49)	63 (46–77)	40 (24–55)	24 (11–40)
Oestrogen receptor	Positive	1398 (82.97)	99 (98–100)	96 (94–97)	93 (91–95)
	Negative	244 (14.48)	86 (81–90)	81 (75–86)	80 (73–85)
Progesterone receptor	Positive	1237 (73.41)	100 (98–100)	97 (95–98)	95 (92–97)
	Negative	401 (23.80)	90 (86–93)	85 (80–88)	81 (76–85)
Her2	Negative	1250 (74.18)	97 (96–98)	93 (92–95)	91 (88–93)
	Positive	294 (17.45)	97 (94–99)	93 (89–96)	89 (84–93)
Intrinsic subtype	Luminal A	997 (59.17)	100 (98–100)	96 (94–98)	94 (92–97)
	Luminal B	331 (19.64)	97 (95–99)	94 (90–97)	88 (83–93)
	Her2	81 (4.81)	94 (86–99)	87 (76–93)	82 (70–91)
	Basal-like	130 (7.72)	78 (70–85)	74 (65–81)	74 (64–82)
	Luminal ONI	91 (5.40)	100 (94–101)	101 (95–102)	101 (92–104)
Grade	I: Well differentiated	329 (19.53)	100 (98–101)	99 (96–101)	99 (95–102)
	II: Moderately differentiated	520 (30.86)	100 (98–101)	95 (92–97)	93 (89–96)
	III: Poorly differentiated	355 (21.07)	93 (89–95)	90 (86–94)	88 (83–92)
	Missing	481 (28.55)	96 (93–98)	91 (88–94)	88 (83–91)

*ONI* Otherwise non-identified, *Luminal ONI* hormonal receptors positive, Her2 missing, *Non-luminal ONI* hormonal receptors negative, Her2 missing

### Tumour characteristics

Regarding the characteristics of the tumours, the most usual histological type was ductal (75.7%), followed by lobular (6.5%). About half of cancers were T1 (52%) and lymph node negatives (52%). Only 42 women (2.5%) had metastasis at the time of diagnosis.

Concerning intrinsic subtypes, 997 (59.2%) could be classified as luminal A-like, 331 (19.6%) as luminal B-like, 81 (4.8%) as Her2 (non-luminal)-like and 130 (7.7%) as basal-like. Grade of differentiation could not be obtained from medical records in 481 patients (28.5%). Almost 31% of the cancers were moderately differentiated while poorly differentiated accounted for about 21% cancers. Hormone receptor status was available for most cases; 83% were oestrogen receptor positive, 73% progesterone positive and 17.4% were Her2 positive (Table 1).

### Relative survival

**Fig. 1:**
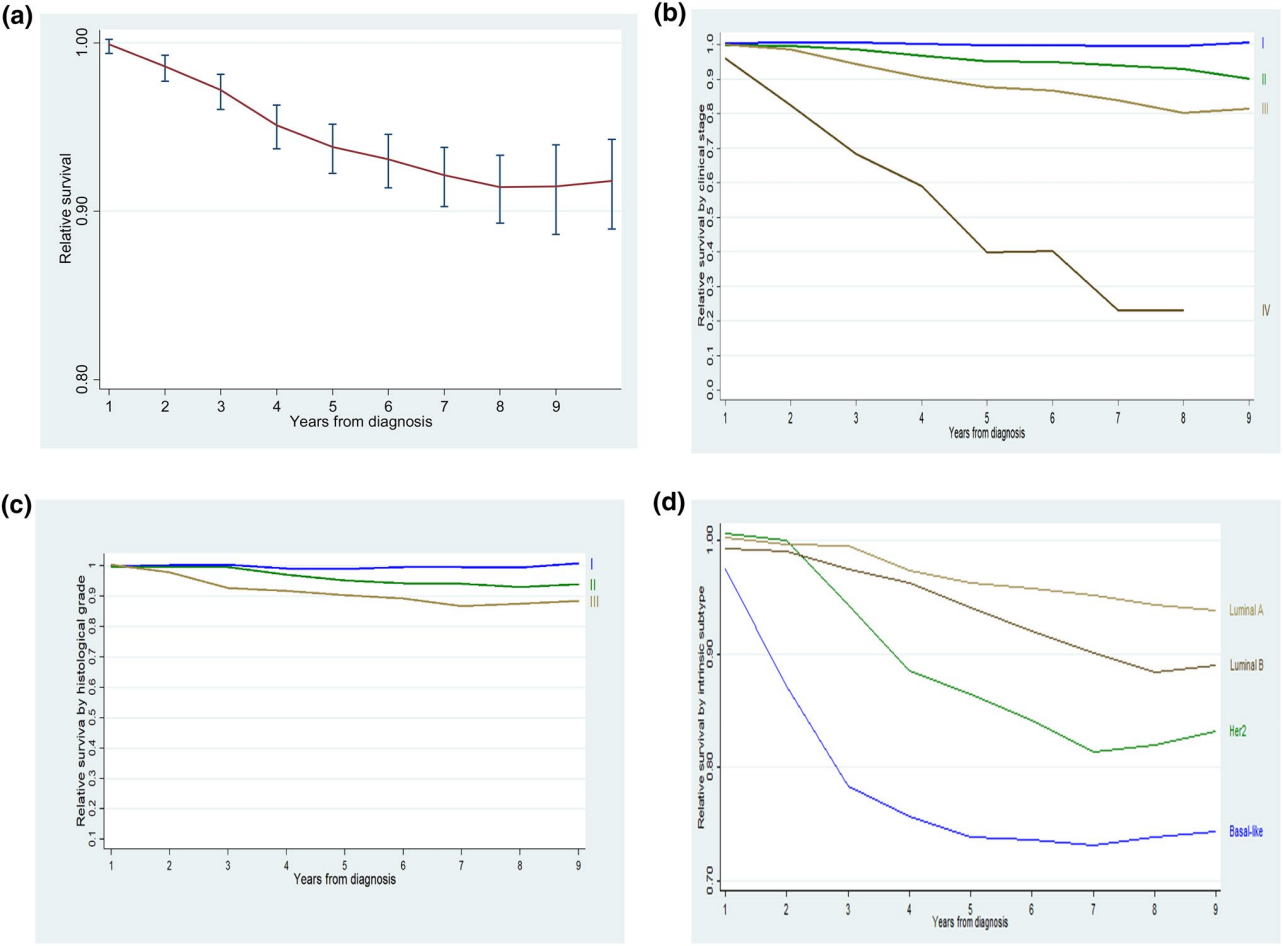
Relative survival in Spanish women with breast cancer: **a** Overall survival, **b** survival according to TNM staging, **c** survival according to grading, **d** survival according to intrinsic subtype

5-year relative survival with breast cancer was 93% (95% CI 92 – 94) (Fig. 1a). Table 1 shows 3-, 5- and 8-year relative survival according to tumour characteristics. Women diagnosed in stage I had the same survival probability than women with the same age without breast cancer (i.e. 100% relative survival) even after 8 years of follow-up. At the same time, relative survival decreased with follow-up time in women diagnosed in more advanced stages: 3-, 5- and 8-year relative survival were 98%, 95% and 93% for breast cancer diagnosed in stage II, 94%, 88% and 81% in women diagnosed in staged III and 63%, 40% and 24% in those diagnosed in staged IV (Table 1 and Fig. 1b). Relative survival also decreased as grading got less differentiated [8-year relative survival: 99% in well differentiated tumours, 93% in moderately differentiated and 88% in poorly differentiated (Table 1 and Fig. 1c)]. Relative survival after 8 years of follow-up was 94% for luminal A-like breast cancers, 88% for luminal B-like, 82% for Her2 non-luminal) and 74% for basal-like cancers (Table 1 and Fig. 1d).

### Prognostic factors on overall mortality

**Fig. 2:**
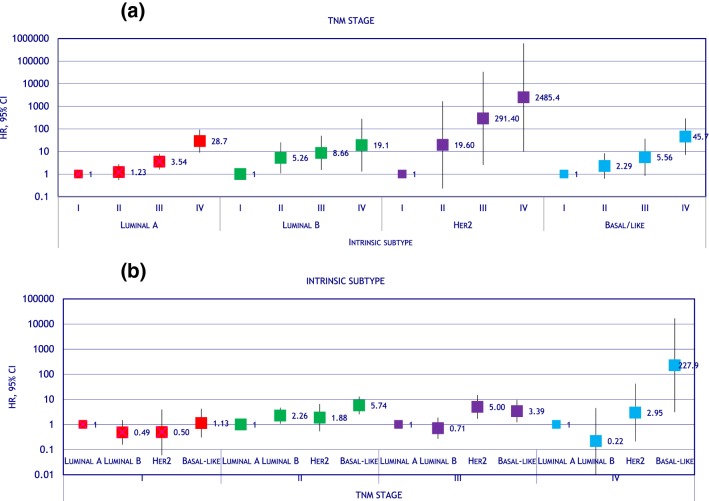
Hazard ratios for overall survival showing the interaction between TNM stage and intrinsic subtype. **a** Hazard ratios for TNM stage stratified by intrinsic subtype, **b** hazard ratios for intrinsic subtype stratified by TNM stage

Age, hospital, stage and grade-adjusted hazard ratios on association between survival and tumour characteristics and first-line treatment are displayed in Table 2. Age, premenopausal status, tumour size, nodal infiltration and presence of metastasis were significantly associated with overall mortality. Hazard ratios increased with tumour stage, being 1 (reference) for T1 and 1.62 (1.04 – 2.52) and 2.04 (1.13 – 3.68) for T2, T3, respectively. Breast cancers negative for oestrogen or progesterone receptors behaved worse than their opposite. Luminal A- like and luminal B-like tumours had similar prognosis; hazard for Her2 (non-luminal) cancers were 50% higher than for luminal A-like (hazard ratio = 1.50; 95% CI 0.87 – 2.59), and basal-like tumour’s hazard was three times that of luminal A-like (hazard ratio = 3.50; 95% CI 2.31 – 5.30). Grading showed a dose–response association with mortality, with hazard ratios 1 (reference) for well differentiated cancers, 1.48 (0.86 – 2.54) for moderately differentiated and 2.42 (1.41 – 4.17) for poorly differentiated. To further explore whether intrinsic subtype adds prognosis value to tumour stage, we studied the effect of stage by stratifying for intrinsic subtype; results in Fig. 2a show that higher stages had higher hazard ratios, whatever the intrinsic subtype. However, when stratifying the effect of intrinsic subtype for tumour stage, we found that intrinsic subtype had no effect at all in patients in stage I, while Her2 (non-luminal) and basal-like tumours had worse prognosis than luminal-like tumours in patients with stage II and, especially, stages III and IV (Fig. 2b).

**Table 2:** Characteristics of the tumour and their association with survival

Variable	Category	*N* (%)	Hazard ratio (95% CI)*	*p*
Histology	Ductal	1276 (75.73)	1 (ref)	
	Lobular	110 (6.53)	1.12 (0.68 to 1.83)	0.666
	Other	299 (17.74)	0.44 (0.26 to 0.72)	0.001
Tumour size	T1	876 (51.99)	1 (ref)	
	T2	424 (25.16)	1.62 (1.04 to 2.52)	0.031
	T3	73 (4.33)	2.04 (1.13 to 3.68)	0.018
	T4	39 (2.31)	1.96 (0.98 to 3.91)	0.056
	Tis	109 (6.47)	0.14 (0.02 to 0.91)	0.04
	Missing	141 (8.37)	1.12 (0.61 to 2.06)	0.718
Node infiltration	N0	877 (52.05)	1 (ref)	
	N1	441 (26.17)	1.16 (0.76 to 1.78)	0.481
	N2	142 (8.43)	1.17 (0.58 to 2.36)	0.651
	N3	49 (2.91)	1.73 (0.81 to 3.72)	0.158
	Missing	176 (10.45)	1.15 (0.65 to 2.03)	0.641
Tumour stage	0	112 (6.65)	0.68 (0.26 to 1.81)	0.444
	I	594 (35.25)	1 (ref)	
	II	498 (29.55)	2.30 (1.46 to 3.61)	> 0.001
	III	187 (11.10)	4.23 (2.60 to 6.88)	> 0.001
	IV	42 (2.49)	29.5 (16.9 to 51.6)	> 0.001
Oestrogen receptor	Positive	1398 (82.97)	1 (ref)	
	Negative	244 (14.48)	2.31 (1.65 to 3.23)	> 0.001
Progesterone receptor	Positive	1237 (73.41)	1 (ref)	
	Negative	401 (23.80)	2.13 (1.58 to 2.87)	> 0.001
Her2	Negative	1250 (74.18)	1 (ref)	
	Positive	294 (17.45)	0.79 (0.55 to 1.14)	0.209
Intrinsic subtype	Luminal A	997 (59.17)	1 (ref)	
	Luminal B	331 (19.64)	1.16 (0.81 to 1.66)	0.429
	Her2	81 (4.81)	1.50 (0.87 to 2.59)	0.143
	Basal-like	130 (7.72)	3.50 (2.31 to 5.30)	> 0.001
	Luminal ONI	91 (5.40)	0.20 (0.05 to 0.82)	0.026
Grade	I: Well differentiated	329 (19.53)	1 (ref)	
	II: Moderately differentiated	520 (30.86)	1.48 (0.86 to 2.54)	0.157
	III: Bad differentiated	355 (21.07)	2.42 (1.41 to 4.17)	0.001
	missing	481 (28.55)	2.31 (1.27 to 4.18)	0.006

Hazard ratios and 95% confidence intervals estimated via Weibull regression

*Hazard ratios adjusted for age, hospital, stage and grade

*ONI* otherwise not identified

#### Pathologic prognostic score in women without neoadjuvant therapy

**Fig. 3:**
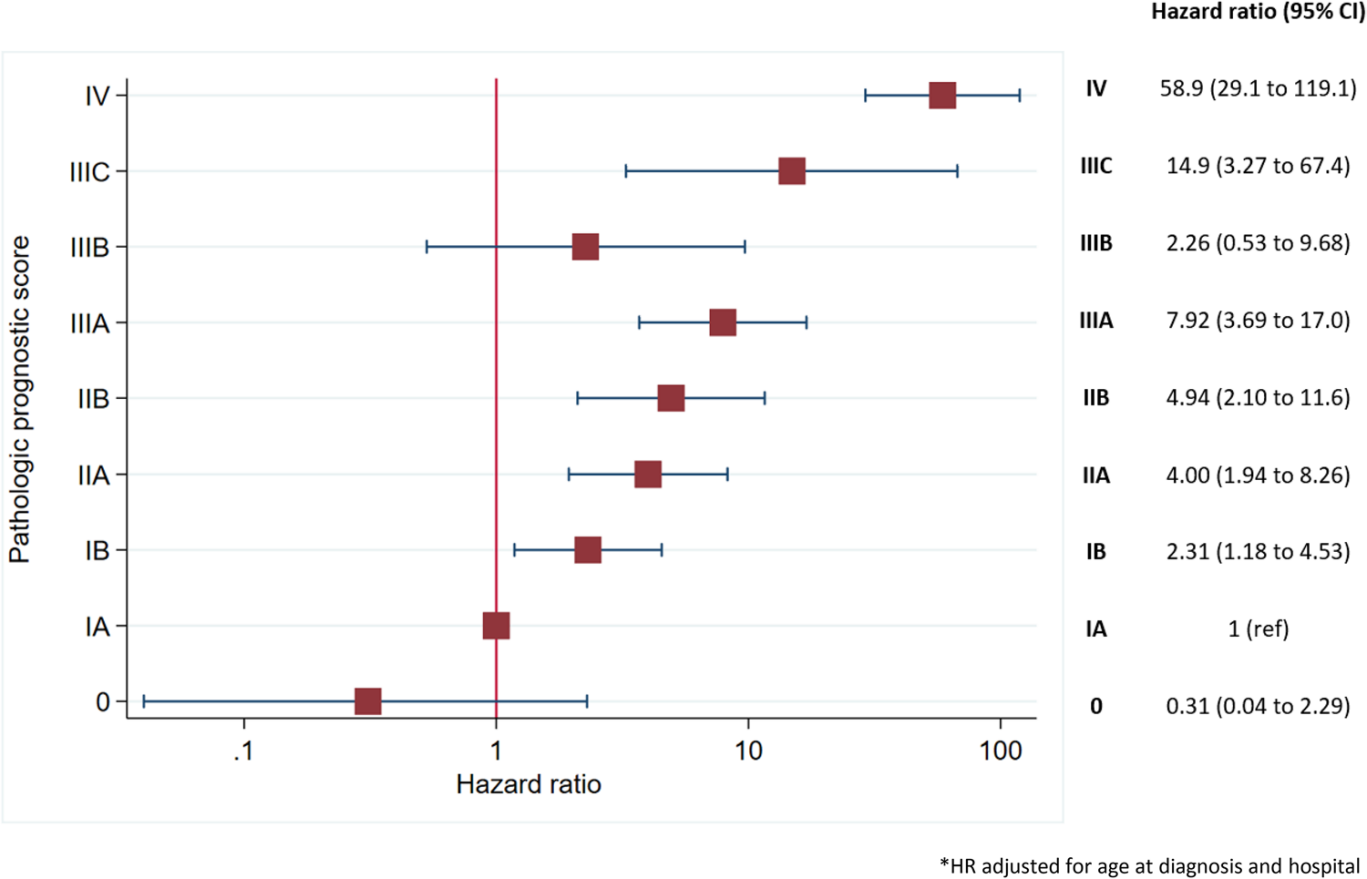
Kaplan–Meier survival estimates according to the AJCC pathological prognostic score

Figure 3 displays the association between the AJCC- Pathologic Prognostic Score and overall survival in women without neoadjuvant therapy. Hazard ratios stepped up as the score rose, being 2.31, 4.00, 4.94, 7.92, 2.26, 14.9 and 58.9 for scores IB, IIA, IIB, IIIA, IIIB, IIIC and IV, respectively. These results are somewhat limited because of their wide confidence intervals. This information appears stratified by menopausal status in supplementary material, where hazard ratios increased faster in premenopausal women (Supplementary Table 2).

#### First-line treatment for patients

Table 3 shows the characteristics of the treatment. Conservative surgery was performed in 1231 (73.1%) patients and mastectomy in the remaining 454 (26.9%). Surgical margins were negative in 66% patients. Radiotherapy was used in 1158 women (68.7%), while adjuvant or neoadjuvant chemotherapy was administered to 50.13% patients. Adjuvant or neoadjuvant hormone therapy was used in 1023 women (60.7%), immunotherapy in 11.1% and Her2-targeted therapy in 152 patients (9.0%).

**Table 3:** First-line treatment and relative survival probability 3, 5 and 8 years after diagnosis

Variable	Category	*N* (%)	3-year	5-year	8-year
			Survival (95% CI)	Survival (95% CI)	Survival (95% CI)
Surgery	Conservative	1231 (73.06)	98 (97–99)	95 (94–97)	93 (91–95)
	Mastectomy	454 (26.94)	94 (91–97)	89 (86–92)	87 (82–91)
Surgical margins	Negative	1104 (65.52)	98 (97–99)	95 (93–97)	94 (91–96)
	Positive	244 (14.48)	100 (97–101)	98 (94–100)	95 (89–98)
	missing	337 (20.00)	92 (88–95)	87 (82–90)	82 (76–87)
Chemotherapy	No	817 (48.49)	98 (97–100)	96 (94–98)	95 (91–97)
	Neoadjuvant	180 (10.68)	95 (90–97)	87 (81–92)	83 (76–88)
	Adjuvant	663 (39.35)	99 (97–100)	96 (94–98)	93 (90–96)
	Palliative	25 (1.48)	40 (21–59)	16 (5–33)	
Radiotherapy	No	334 (22.45)	95 (92–98)	91 (87–95)	88 (81–93)
	Neoadjuvant	5 (0.34)	61 (13–89)	21 (1–60)	
	Adjuvant	1128 (75.81)	100 (99–100)	97 (96–98)	95 (93–97)
	Palliative	21 (1.41)	31 (13–52)	16 (4–35)	
Immunotherapy	No	1498 (88.90)	98 (97–99)	95 (93–96)	93 (90–95)
	Neoadjuvant	27 (1.60)	90 (70–97)	75 (54–88)	67 (45–83)
	Adjuvant	144 (8.55)	98 (94–100)	95 (89–98)	92 (85–97)
	Palliative	16 (0.95)	41 (17–64)	28 (9–51)	
Endocrine therapy	No	662 (39.29)	94 (91–96)	90 (87–92)	86 (82–90)
	Neoadjuvant	19 (1.13)	103 (103–103)	76 (48–93)	72 (40–94)
	Adjuvant	985 (58.46)	100 (99–101)	98 (97–99)	97 (94–99)
	Palliative	19 (1.13)	61 (35–80)	26 (9–49)	

Hazard ratios on association between survival and first-line treatment are displayed in Table 4. Type of surgery (mastectomy/conservative surgery) and surgical margins were not associated with survival. Among systemic treatments, adjuvant therapy (whether chemo-, radio- or hormone-therapy) was associated with lower mortality and both neoadjuvant and palliative treatment were associated with worse prognosis (Table 4).

**Table 4:** First-line treatment and their association with survival

Variable	Category	*N* (%)	Hazard ratio (95% CI) *	*p*
Surgery	Conservative	1231 (73.06)	1 (ref)	
	Mastectomy	454 (26.94)	0.83 (0.56 to 1.22)	0.339
Surgical margins	Negative	1104 (65.52)	1 (ref)	
	Positive	244 (14.48)	0.84 (0.50 to 1.42)	0.518
	Missing	337 (20.00)	1.03 (0.67 to 1.56)	0.905
Chemotherapy	No	817 (48.49)	1 (ref)	
	Neoadjuvant	180 (10.68)	1.18 (0.72 to 1.91)	0.514
	Adjuvant	663 (39.35)	0.60 (0.40 to 0.89)	0.010
	Palliative	25 (1.48)	8.44 (4.61 to 15.4)	< 0.001
Radiotherapy	No	334 (22.45)	1 (ref)	
	Neoadjuvant	5 (0.34)	6.05 (1.88 to 19.5)	0.003
	Adjuvant	1128 (75.81)	0.44 (0.30 to 0.65)	< 0.001
	Palliative	21 (1.41)	5.31 (2.57 to 11.0)	< 0.001
Immunotherapy	No	1498 (88.90)	1 (ref)	
	Neoadjuvant	27 (1.60)	1.79 (0.87 to 3.67)	0.113
	Adjuvant	144 (8.55)	0.83 (0.48 to 1.44)	0.518
	Palliative	16 (0.95)	5.22 (2.57 to 10.6)	< 0.001
Endocrine therapy	No	662 (39.29)	1 (ref)	
	Neoadjuvant	19 (1.13)	1.17 (0.50 to 2.76)	0.719
	Adjuvant	985 (58.46)	0.51 (0.37 to 0.71)	< 0.001
	Palliative	19 (1.13)	2.18 (1.05 to 4.56)	0.037

Hazard ratios and 95% confidence intervals estimated via Weibull regression

*Hazard ratios adjusted for age, hospital, stage and grade

## Discussion

In spite of recent improvements in breast cancer systemic therapy, our results confirm that tumour stage and grading are major prognosis factors regarding overall survival with breast cancer. Among components of TNM staging system, tumour size greater than 50 mm (i.e. T3 or T4) more than doubled the risk of dying, while N3 nodal involvement and presence of metastasis had a huge effect on mortality. The pathological prognostic score suggested by the AJCC in 2017 combining anatomic characteristics (i.e. TNM staging) with biological characteristics (i.e. grading and hormonal/Her2 receptors) strongly correlated with survival.

TNM staging has largely been the main factor regarding survival with breast cancer, which led the AJCC to suggest its Anatomic Stage Groups [11]. Our results on TNM components are consistent with recent studies still proving that TNM classification is a main contributor to survival [10, 23–25]. For instance, a large study with data from Netherlands Cancer Registry reported hazard ratios over 2.5 for T3 or T4 tumours and 4.0 for N3 tumours in women diagnosed between 2006 and 2012. These hazard ratios were similar to those obtained in the same registry for women diagnosed between 1999 and 2005 [10], which reinforces TNM staging relevance for survival has not changed with recent treatment improvements, thus strengthening the importance of early diagnosis.

Recent developments, however, have focused on tumour biology, making the identification of breast cancer subtypes via differentiation grade or presence of hormonal and Her2/neu receptors possible, heading to specific therapies according to tumour histological and molecular characteristics [6, 26–28]. Our results also confirmed higher survival for women with breast cancer positive to either oestrogen or progesterone receptors and negative to HER2 receptors, as well as worsening prognostic as grading increased from well to poorly differentiated tumour. In this regard, the new AJCC Pathological Prognostic Score [11] has been suggested by combining both anatomic staging and biological markers, thus refining the previous AJCC Anatomic Stage Groups [12], which is now intended only for regions were biological markers are not routinely available [11]. In our cohort, the AJCC Pathological Prognostic Score had large prognostic value with hazard ratios swiftly rising as the Pathological Prognostic Score increased. In addition, this score presents a higher predictive ability in premenopausal women. This can be explained by (i) the rate at which the cancer develops—tumours could be faster and receive poorer prognosis in premenopausal women—and (ii) the fact that a younger woman, i.e. premenopausal, with breast cancer is more likely to die because of the tumour itself, as opposed to postmenopausal women, who have more chances to die due to other causes. Of note, the analysis of Pathological Prognostic Score should have been restricted to women without neoadjuvant therapy as this score is based on the pathological TNM staging, which is obtained after surgery.

Adjuvant chemotherapy, radiotherapy or endocrine therapy was associated with better prognosis in our cohort, which agrees with previous reports [4, 29–31]. Neoadjuvant therapy, however, was associated with lower survival. Results on systemic therapy—survival relationship cannot be straight forwardly interpreted as, even after adjusting for stage and grade, there is still room for confounding by indication as women with more severe cancer are more likely treated with more aggressive therapies. For instance, neoadjuvant therapy is not usually recommended for low proliferating cancers and hormone receptor-positive, HER2-negative, grading I–II cancers [26]; therefore, its high hazard ratios could be due to residual confounding as it is frequently indicated in cancers with worse prognostic.

Our study has some limitations. Firstly, the sample size—although enough for general results—is small for studying specific subgroups or interactions. Secondly, basal information was obtained from medical records, which could have partial information on some variables, leading to some amount of missing data. On this subject, it is noteworthy that missing categories did not follow a clear pattern in our results as missing data in TNM components behaved almost as the reference category (i.e. good prognostic), while missing data in grading performed as poorly differentiated tumours. Therefore, it is not possible for us to attribute any data omission in medical records to its irrelevance. Thirdly, our sample could be biased towards cancers with better prognostic as women with advanced disease could have refused to participate, thus underrepresenting women in stage IV; had this occurred, it could have overestimated relative survival; however, this problem should have not biased hazard ratios estimates. Finally, the multicentric design of our study is both a limitation and a strength. A limitation as routine clinical practice could vary from hospital to hospital, eventually increasing random variation; we dealt with this problem by adjusting all our analyses for hospital. Nevertheless, it is also a strength as it obtained a fair representation of usual clinical practice in Spain about 2008–2013. An additional strength is the active prospective follow-up, carried out via three complementary sources: medical records, phone calls to the patient and National Death Index consultation.

## Conclusions

In conclusion, both TNM staging and histological/molecular biomarkers are associated with overall survival in Spanish women with breast cancer; when both are combined in the AJCC Pathological Prognostic Score, its prognostic value vastly improved in women without neoadjuvant therapy.

## Electronic supplementary material

Below is the link to the electronic supplementary material.

Supplementary file 1—Supplementary Figure 1. Relative survival with breast cancer according to components of TNM staging.(a) Tumour size, (b) nodal involvement, (c) metastasis. Supplementary Figure 2. Relative survival with breast cancer according to receptors positivity. (a)Oestrogen receptors, (b) progesterone receptors, (c) Her2 receptors. (PDF 199 kb)

## Abbreviations

BC: Breast cancer
BMI: Body mass index
MCC-Spain: Multicase-control Spain
OR: Odds ratios
RRR: Relative risk ratio
CI: Confidence intervals
