# Supplementary material for: Tumour characteristics and survivorship in a cohort of breast cancer: the MCC-Spain study
Source: Breast Cancer Res Treat. 2020 Apr 30;181(3):667–78. doi: 10.1007/s10549-020-05600-x (PMC7220874; doi:10.1007/s10549-020-05600-x)
Supplement: Supplementary file 1 — Supplementary Figure 1. Relative survival with breast cancer according to components of TNM staging.(a) Tumour size, (b) nodal involvement, (c) metastasis. Supplementary Figure 2. Relative survival with breast cancer according to receptors positivity. (a)Oestrogen receptors, (b) progesterone receptors, (c) Her2 receptors. (PDF 199 kb) [file 10549_2020_5600_MOESM1_ESM.pdf]

**Supplementary table 1. Main characteristics of the patients. Sample description.**

| Variable | Category | N(%) |
|---|---|---|
| Age | (mean±sd) | 56.5(12.6) |
| Age | <45 years | 352(20) |
| | 45 – 54 years | 456(26) |
| | 55 – 64 years | 463(27) |
| | 65 – 74 years | 289(17) |
| | ≥75 years | 178(10) |
| Educational level | Less than primary school | 270(16) |
| | Primary school | 563(32) |
| | Secondary school | 574(33) |
| | University | 331(19) |
| Menopausal status | Premenopausal | 612(35) |
| | Postmenopausal | 1125(65) |
| Smoking | Never smoker | 970(56) |
| | Former smoker | 286(17) |
| | Current smoker | 469(27) |
| Body Mass Index ($kg/m^2$) | <18.5 | 28(1.7) |
| | 18.5 – 24.9 | 745(46) |
| | 25.0 – 29.9 | 542(34) |
| | ≥30.0 | 293(18) |

**Supplementary table 2. Pathologic prognostic score relation with survival in women without neoadjuvant therapy by menopausal status. Hazard ratios and 95% confidence intervals estimated via Weibull regression**

| | Premenopausal | | | Postmenopausal | | |
|---|---|---|---|---|---|---|
| **PPS** | **N (%)** | **Hazard ratio (95% CI)*** | **p** | **N (%)** | **Hazard ratio (95% CI)*** | **p** |
| **0** | 23 (5.64) | NA | NA | 60(9.54) | 0.42 (0.06 to 3.26) | 0.410 |
| **IA** | 226(55.39) | 1 (ref) | | 367(58.35) | 1 (ref) | |
| **IB** | 66(16.18) | 3.18 (0.55 to 18.5) | 0.198 | 82(13.04) | 2.34 (1.01 to 5.43) | 0.047 |
| **IIA** | 31(7.60) | 8.42 (1.55 to 45.7) | 0.014 | 40(6.36) | 3.08 (1.18 to 8.06) | 0.022 |
| **IIB** | 15(3.68) | 5.20 (0.47 to 57.1) | 0.178 | 29(4.61) | 3.67 (1.23 to 11.0) | 0.020 |
| **IIIA** | 25(6.13) | 18.3 (3.28 to 101.8) | 0.001 | 18(2.86) | 5.45 (1.87 to 15.9) | 0.002 |
| **IIIB** | 6(1.48) | 13.7 (1.29 to 145.2) | 0.030 | 13(2.07) | NA | NA |
| **IIIC** | 2(0.49) | NA | NA | 4(0.64) | 11.5 (1.17 to 113.6) | 0.036 |
| **IV** | 14(3.43) | 139.3 (24.3 to 799.0) | 0.000 | 16(2.54) | 39.6 (14.8 to 105.9) | 0.000 |

*Adjusted for age at diagnosis and hospital

NA = Not Available/Not Applicable

Sup. Fig. 1

(a)

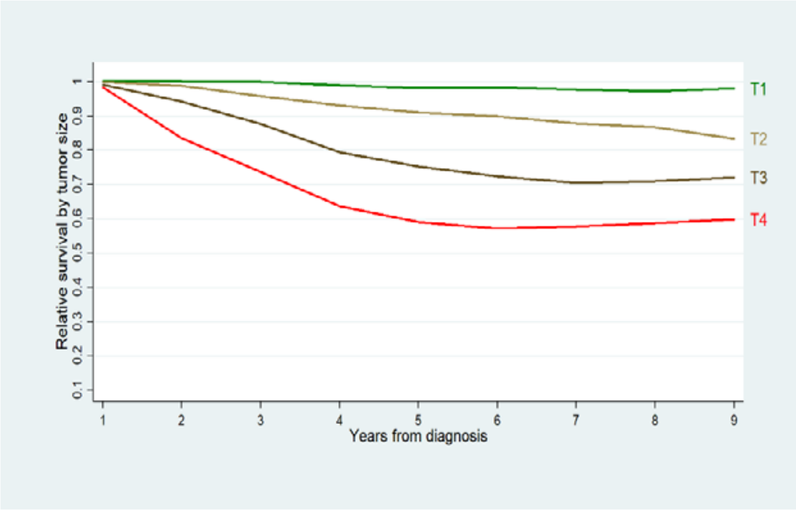

Relative survival by tumor size
Years from diagnosis
T1
T2
T3
T4


(b)

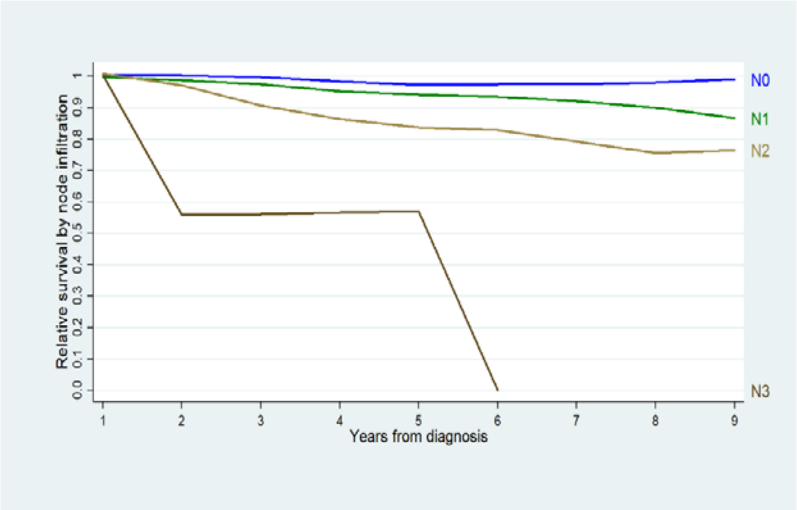

Relative survival by node infiltration
Years from diagnosis
N0
N1
N2
N3


(c)

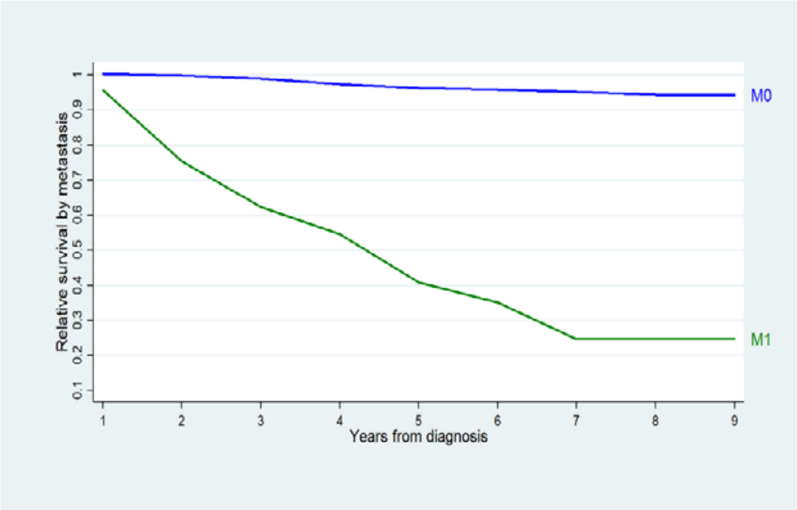

Relative survival by metastasis
Years from diagnosis
M0
M1

Sup. Fig. 2

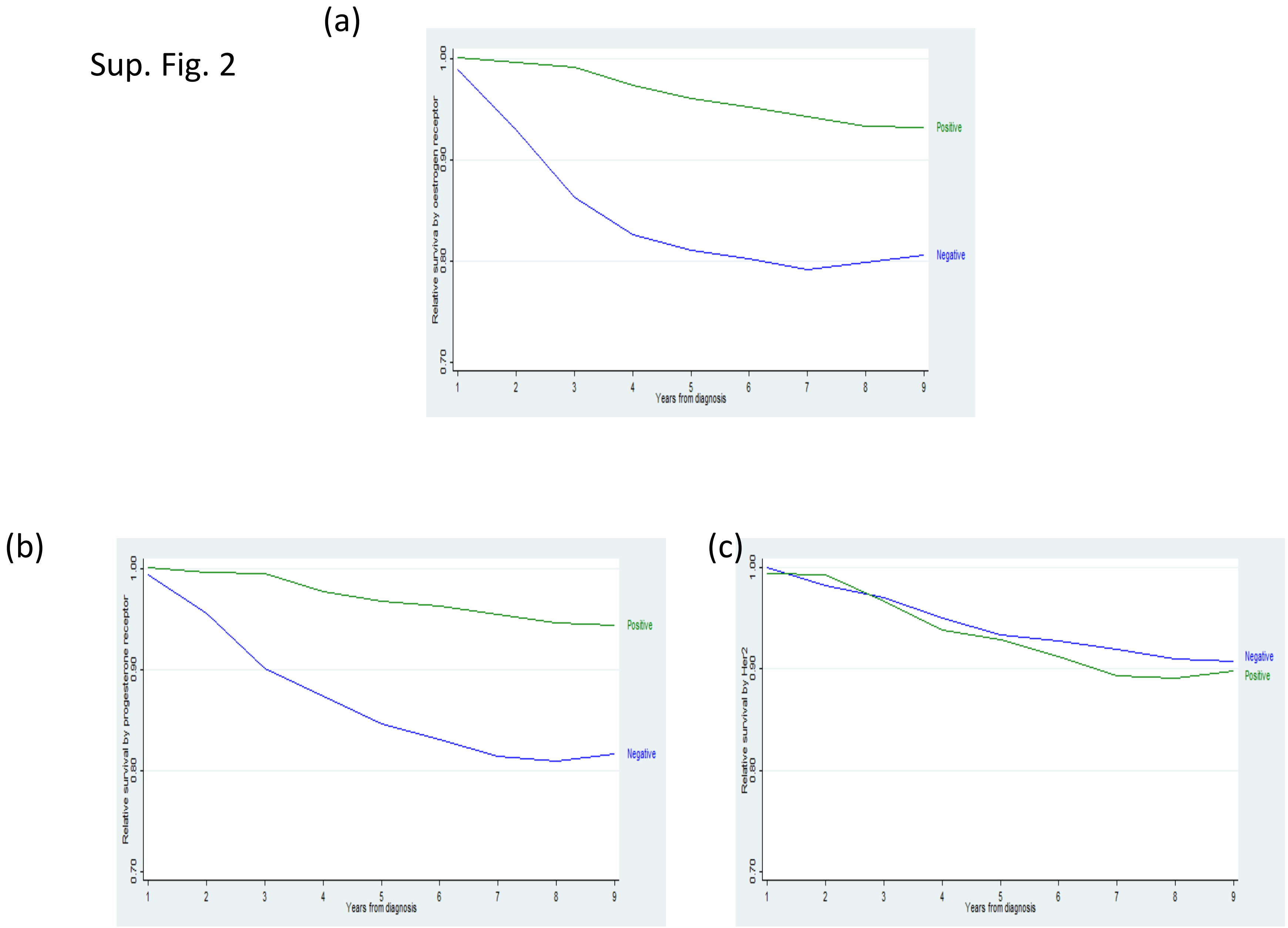
(a)
Relative surviva by oestrogen receptor
1.00
0.90
0.80
0.70
Positive
Negative
1
2
3
4
5
6
7
8
9
Years from diagnosis
(b)
Relative survival by progesterone receptor
1.00
0.90
0.80
0.70
Positive
Negative
1
2
3
4
5
6
7
8
9
Years from diagnosis
(c)
Relative survival by Her2
1.00
0.90
0.80
0.70
Negative
Positive
1
2
3
4
5
6
7
8
9
Years from diagnosis
